# Hospitals and State Insurance

**Published:** 1911-06-10

**Authors:** 


// The Hospital

A Journal of

The Medical Sciences and Hospital Administration.

New Series. No. 228, Vol. IX [No. 1300, Vol. L.] SATURDAY, JUNE 10, 1911

### Hospitals and State Insurance.

The special conference convened by the British Hospitals Association last week to discuss the points in the Government Insurance scheme so far as they concern voluntary charitable institutions in this country is a step in the right direction. We sincerely trust that the expression of authoritative opinion, emanating from this conference, will be appreciated in the proper quarter, and that the representations of hospital workers will receive due consideration from those who are responsible for the ultimate text of the Bill. We have repeatedly urged hospital workers to study this important question and to make their views known. The interest which has been aroused, and the instructive manner in which the scheme has been criticised, are reasons to warrant the belief that hospital authorities will see to it that their opinions are pressed home and made practically effective by co-operation and unanimous action. A close study of the effect which the Insurance Acts have had upon the hospitals in Germany is to be commended to hospital workers in this country. We hope soon to publish a series of special articles dealing with the subject so far as it affects hospitals and voluntary charitable organisations. Meanwhile we wish to draw our readers' attention to two very interesting and important works on the subject which deal very fully with the whole question of State Insurance in Germany. The one is Keidel's "Invalidenversicherung," and the other, the same author's "Krankenversicherung" both of which give details of the texts of the German Acts, and, what is of far greater importance to hospital workers at the present moment, summaries of the legal decisions on debatable points in the Acts. There have recently been many instructive expressions of opinion emanating from German legal authorities on such points as the insurance of nurses employed in hospitals, of hospital servants, members of the hospital staff, and also on the special points with regard to the right of the insured to claim hospital treatment in certain cases. This last point is of special interest to us, since in the German Act no provision is made for hospital treatment beyond the bare outlined suggestion contained in Section I. of the revision of April 1892. The question with reference to the support to be given to such institutions as take in members of friendly and sick societies has been fully discussed, and the legal decisions on the point are perfectly clear. We were told last week by the Chancellor of the Exchequer that one of the effects of State Insurance in Germany has been an increase in the donations, and thus in the voluntary income of the German hospitals. A close study of the facts does not bear out that contention. It is true the income of the hospitals has increased, but such increase is due to the fact that the proportionate payments from the "Krankenkassen" have risen owing to the proportionately larger number of members treated year by year. A glance at the figures for twenty-six hospitals which we have before us confirms this.

The probable effect of the Government scheme, at least in the immediate future, have been clearly pointed out by writers in the columns of The Hospital as well as by speakers at last week's conference. We have no reason to believe that these views are exaggerated fears on the part of hospital workers; on the contrary, we think that they are fully warranted by facts, and we sincerely trust that hospital authorities will continue to press them until such modifications are made in the text of the Bill as will ensure the continuance of that excellent work which is now being done by the voluntary hospitals in this country. There are many other points, apart altogether from this question of finance, which affect the hospitals very closely so far as this measure is concerned. Some of them have already been fully ventilated; others we hope to deal with during the ensuing weeks. It is imperative that the provisions of the Bill should be clear and unequivocal, especially with regard to the definition of those who are entitled to the benefits of insurance. Mr. George's lucid and straightforward explanations during the past week, and the full discussion on the second reading of the Bill, have greatly tended to clear the ground, but there are still some points on which the hospital worker, and those who are interested in the existence of the voluntary system, would like to have some further light. We welcome the Bill, as we have said more than once, as a decided advance towards the improvement of social conditions in this country, but at the same time we desire that it should be calmly and thoroughly subjected to the most searching criticism possible.

Only in that way can it be made thoroughly practical and efficient. It would be an unfortunate thing for the community if it should prove necessary to amend the Act in a year's time. The German example here ought to be a lesson and a warning to us. With that example before us we are in a position to appreciate the danger of attempting to rush through so important and far-reaching a measure as this without careful thought and adequate discussion. If State Insurance is to be of real help to us, it must be designed in such a manner that it will aid, instead of hamper, the existing movements for the amelioration of social conditions; it must act in co-operation with the best that is already in being, instead of calling into life other forces and other movements of whose ultimate effects we are as yet unadvised. For several centuries the hospitals in this country have borne the brunt of the treatment of the necessitous sick and injured. They have established their right to exist through the good work that they have achieved, and have shown that they are capable, even under the altered and highly complex conditions of modern life, to continue their work with efficiency and thoroughness unsurpassed by any other voluntary institutions in the world. If they are to be saddled with more work, they must see to it that they get an equivalent benefit in return. For that reason we would once more lay stress on the urgent desirability of studying the question of State Insurance very closely, and of voicing freely any criticism, whether destructive or constructive, so that the public and the Government may be under no misapprehension as to what the hospitals desire and in what manner the Bill may be made to safeguard interests which are not those of a class merely but of the community at large.

